# Enhancing clinical interpretation of patient‐reported outcomes: A cross‐platform toolkit for clinically important difference estimation using R Shiny and statistical analysis system

**DOI:** 10.1002/gps3.70041

**Published:** 2026-08-13

**Authors:** Zongshi Qin, Yongpei Yu, Kuan Liao, Dongdong Shi, Zhen Wang, Hongqiu Gu, Jiani Wu

**Affiliations:** ^1^ Center for Digital Health and Artificial Intelligence Peking University First Hospital Beijing China; ^2^ Clinical Research Institute Institute of Advanced Clinical Medicine Peking University Beijing China; ^3^ JC STEM Lab of Digital Oncology Care Enhancement (DOCE) The Hong Kong Jockey Club Charities Trust Hong Kong China; ^4^ Shanghai Mental Health Center Shanghai Jiao Tong University School of Medicine Shanghai China; ^5^ Department of Neurology Beijing Tiantan Hospital Capital Medical University China National Clinical Research Center for Neurological Diseases Beijing China; ^6^ Department of Acupuncture Guang'anmen Hospital China Academy of Chinese Medical Sciences Beijing China; ^7^ Department of Psychiatry Massachusetts General Hospital Harvard Medical School Charlestown Massachusetts USA

**Keywords:** clinically important difference, patient‐reported outcome measure, R Shiny, SAS

## Abstract

**Objective:**

Estimating clinically important differences (CIDs) for patient‐reported outcome measures (PROMs) is essential for translating statistical findings into clinically meaningful treatment effects. We developed estimating clinically important differences (ESTICID), a cross‐platform toolkit implemented in both R Shiny and statistical analysis system, to estimate and visualise CIDs in longitudinal studies.

**Methods:**

ESTICID applies an anchor‐based approach using linear mixed‐effects models with random intercepts. The framework accommodates repeated measures, supports both continuous and categorical anchors and handles incomplete follow‐up data under a missing‐at‐random assumption. A multicenter randomised controlled trial dataset was used for illustration.

**Examples:**

Using the illustrative dataset, we show that ESTICID generates clinically interpretable CID estimates across different levels of improvement and provides graphical outputs that facilitate interpretation. In simulation analyses, the estimates remained stable under increasing levels of missing follow‐up data. The R Shiny implementation further enables interactive, browser‐based use without programming.

**Conclusions:**

ESTICID provides a practical and accessible framework for anchor‐based CID estimation in longitudinal PROM research. By integrating methodological rigour with cross‐platform implementation, it may improve the interpretation of treatment effects, responder definitions, sample size estimation and evidence‐to‐decision processes in psychiatry and related clinical fields.

## Abstract (highlights)

**WHAT IS ALREADY KNOWN ON THIS TOPIC**

- Estimating clinically important differences (ESTICID) estimates both CID_within‐group_ and CID_between‐group_ for patient‐reported outcome measures using an anchor‐based mixed‐effects modelling framework.

**WHAT THIS STUDY ADDS**

- The toolkit conceptualises CID as a spectrum of clinical importance, including minimal, moderate and marked categories, rather than a single universal cutoff.

**HOW THIS STUDY MIGHT AFFECT RESEARCH, PRACTICE OR POLICY**

- By providing both an R Shiny application and a statistical analysis system macro, ESTICID lowers technical barriers to clinically meaningful interpretation of patient‐reported outcome measures and may support responder definition, trial interpretation, guideline development and sample size estimation.

## INTRODUCTION

In clinical research, patient‐reported outcomes (PROs) are widely used to evaluate symptoms, functioning and health‐related quality of life.^1^, ^2^ Statistical significance alone, however, does not indicate whether an observed change is meaningful to patients or whether the intervention‐placebo difference is clinically relevant.^3^, ^4^ Statistical significance can be achieved for tiny differences, but this finding does not indicate whether patients have experienced meaningful clinical benefits or treatment effects.^5^ For this reason, the estimation of clinically important differences (CIDs) has become essential for translating patient‐reported outcome measure (PROM) results into clinically interpretable information.^6^, ^7^

CID represents a generalised framework that captures the full spectrum of clinically meaningful effects, including varying magnitudes, bidirectional changes and both within‐group and between‐group comparisons.^8^ Unlike purely statistical significance, CID functions as a latent threshold of perception, a value that exists independently of the statistical methods used to estimate it. However, the ‘minimal’ CID cannot meet all needs in medical decision‐making. The moderate or large change or difference is also important in data interpretation and clinical decision‐making.^9^ This assessment should be explicit and include considerations around the probability, magnitude and importance of health‐related benefits, harms and other desirable and undesirable consequences of the recommendation or decision.^10^, ^11^ Traditionally, clinimetric studies focus on within‐group minimal changes, estimating how much an individual or a group improves from baseline to the end of treatment.^3^, ^12^, ^13^ This kind of estimation, sometimes arbitrarily referred to as minimal important change (MIC) or minimal important difference (MID),^3^ is valuable for interpreting individual‐level improvement. In clinical trials, particularly randomised controlled trials (RCTs) involving a control group, the most informative estimate is the between‐group difference, regardless of whether this effect size is small, moderate or large, reflecting the magnitude of the difference in meaningful change attributable to treatment, beyond natural progression or placebo effects.^7^ Also, the between‐group difference is critical for prospective sample size estimation, non‐inferiority margin setting and the treatment efficacy judgement.^14^, ^15^ To clearly clarify clinically important within‐group difference and clinically important between‐group difference, we herein propose the concept of CID_within‐group_ and CID_between‐group_.^8^ Whilst CID_within‐group_ reflects what matters to a group of patients, the CID _between‐group_ reflects what matters to a clinical decision: whether the intervention makes a clinically meaningful difference compared to the control. This distinction is particularly emphasised in regulatory and health technology assessment contexts, where CID_between‐group_ serves as a benchmark for efficacy claims, but CID_within‐group_ serves as responder definitions. Furthermore, CID is conceptualised as a spectrum of clinical significance encompassing minimal, moderate and marked categories, providing a flexible reference range rather than a rigid cutoff for decision‐making frameworks.

Among the various methods used to estimate either CID_between‐group_ or CID_within‐group_, the anchor‐based approach remains a gold standard due to its interpretability and direct connection to patient‐perceived changes.^16^ Anchors such as the Patient Global Impression (PGI) or Clinician Global Impression are often used to classify patients into meaningful categories (e.g., improved, unchanged, worsened), enabling estimation of CID in a clinically intuitive way.^5^, ^17^, ^18^, ^19^ Despite its advantages, anchor‐based CID estimation is not straightforward in implementation. It requires careful anchor selection, category definition, handling of missing data and consideration of whether CID should be interpreted at the within‐group or between‐group level. However, available statistical tools do not provide flexible, automated support for both levels of analysis. A current statistical analysis system (SAS) macro could be used to estimate CIDs for both between‐group and within‐group analyses, using the receiver operating characteristic (ROC) curve method and automatically identifying the cut‐off point for CID.^20^ However, ROC‐based methods require a dichotomous anchor and are less straightforward to apply when anchors have multiple ordered categories or when data are unbalanced and longitudinal, whereas mixed‐effects models naturally accommodate repeated measures. To address this gap, we developed a user‐friendly toolkit for estimating clinically important difference (ESTICID) that can estimate and visualise both CID_within‐group_ and CID_between‐group_ using an anchor‐based approach based on a mixed‐effects model and made it available via R and SAS. The ESTICID accommodates longitudinal data for incomplete data conditions, supports CID estimation across various categories and produces clinically interpretable outputs. This cross‐platform toolkit will enhance methodological transparency, reproducibility and relevance to clinical research.

## METHODS

### Conceptual overview of CIDs and anchor‐based approach

A CID_within‐group_ refers to a change in a PROM that is perceived by patients as clinically meaningful, whether representing improvement or worsening. Anchor‐based approaches establish CID thresholds by linking changes in PROM scores to an external reference (or ‘anchor’) that reflects the patient's overall perception of change. In practice, PROMs are measured both at baseline and after treatment. Anchors, such as the Patient Global Impression of Change (PGI‐C), are typically assessed post‐treatment, whilst others, like the PGI‐S (status), may also be collected at baseline. The anchor serves as an external indicator to interpret the magnitude of change in PROMs. CID_between‐group_ estimation follows a similar concept but compares the mean change in PROMs between anchor‐defined groups. Specifically, it contrasts the target group (e.g., ‘minimally improved’) with a reference group, usually defined as ‘no change’. The anchor must be at least moderately correlated with the PROM to ensure its clinical relevance. In this study, we apply a within‐group anchor‐based approach: patients are categorised based on their PGI‐C responses and the mean PROM change is estimated within each anchor‐defined group. The average change in the ‘small improvement’ category (e.g., ‘somewhat better’) is commonly taken as the within‐group CID, with higher categories representing larger, more meaningful improvements.

### Mixed‐effects model for estimation of CIDs

To estimate CIDs in longitudinal PROMs, we applied an anchor‐based approach using linear mixed‐effects models (LMMs). All analyses were conducted using a custom SAS macro program, ESTICID, developed to automate estimation, visualisation and classification of CIDs across anchor‐defined groups. For each subject, changes in PROM scores from baseline were calculated. The anchor variable (e.g., PGI‐C) was incorporated either as a continuous covariate or as a categorical factor, depending on user specification. An LMM with a subject‐specific random intercept was fitted to account for repeated measurements and within‐subject correlation over time. The general form of the model is as follows:

$$\Delta {\text{PROM}}_{ij}=\beta_{0}+f({\text{Anchor}}_{ij})+u_{i}+\varepsilon_{ij},$$

where

- ΔPROM_*ij*_, the change in PROM score for subject ***i*** at time point ***j***, calculated as the difference from baseline (PROM_*ij*_ − PROM_*i*0_);
- *β*_0_, fixed intercept representing the expected change when anchor equals 0 (or reference category);
- *f*(Anchor_*ij*_), a flexible function of the anchor variable. If the anchor is modelled as a continuous variable, *f*(Anchor_*ij*_) = *β*1⋅Anchor_*ij*_; If the anchor is categorical, *f*(Anchor_*ij*_) = $\sum_{k=1}^{K}\beta_{k}\cdot D_{k,ij}$, where $D_{k,ij}$ are dummy variables representing anchor levels and *K* is the number of non‐reference levels;
- *u*_*i*_ ∼ *N* (0, *σ*_*u*_^2^), random intercept for subject *i*, accounting for between‐subject variability;
- *ϵ*_*ij*_ ∼ *N* (0, *σ* ^*2*^), within‐subject residual error term.

This model structure allows the macro to flexibly accommodate both types of anchor variables, enabling the estimation of adjusted PROM changes across anchor‐defined subgroups or along a continuous anchor gradient. The choice of modelling the anchor as categorical or continuous is specified by the user via the macro parameter ‘categoric_anchor’.

### Data structure and processing scenarios in ESTICID

ESTICID should be applied when the PROM is measured at baseline and at least one follow‐up assessment, when the anchor is clinically interpretable and directionally ordered and when the anchor demonstrates sufficient association with PROM change to support anchor‐based interpretation. Two typical data scenarios are supported by the ESTICID, depending on how the anchor and PROM variables are measured and structured. Scenario 1: The PROM change score is modelled as the independent variable and the anchor (e.g., PGI‐C) serves as the dependent variable. In this case, the anchor is assessed only after treatment and no baseline measurement is available (Supplemental File 1). The macro automatically assumes a baseline anchor value of zero to calculate change scores. Scenario 2: The PROM remains the independent variable, whilst the anchor (e.g., PGI‐S) is treated as the dependent variable. In this setting, both baseline and follow‐up values of the anchor are available. The macro computes the change in anchor values by subtracting the baseline value from the post‐treatment value. For both scenarios, the PROM change from baseline is computed internally by the macro. Users must provide a longitudinal dataset containing subject ID, time point, PROM scores and anchor values (either raw or post‐treatment only). The macro handles missing baseline anchors through imputation (assuming zero) and excludes baseline rows from model fitting to avoid bias in estimating treatment‐related change.

(1) *Sorting and baseline identification*: The data are first sorted by subject ID and time. For each subject, the baseline PROM and anchor scores (i.e., at the first time point) are identified and merged onto all follow‐up records.
(2) *Calculation of PROM change scores*: A new variable, chg_prom, is created to represent the change in PROM scores from baseline at each follow‐up visit.
(3) *Anchor coding and transformation*: If the anchor is a global impression of change (e.g., PGI‐C), the macro uses the anchor directly as the outcome variable in modelling. If the anchor reflects current status (e.g., PGI‐I or PGI‐S), a new variable, chg_anchor, is computed as the change from baseline for use in the model.
(4) *Correlation diagnostics*: Before fitting the model, the macro optionally calculates the Pearson correlation coefficient (and associated *p*‐value) between the anchor (treated as an ordinal variable) and chg_prom. This helps assess whether the anchor is sufficiently correlated with the PROM to justify its use. If the correlation is weak or non‐linear, the anchor is automatically treated as a categorical variable in the model.
(5) *Mixed‐effects modelling*: An LMM is fitted using the PROC MIXED procedure. The primary model includes chg_prom as the dependent variable and the anchor (continuous or categorical) as the main predictor. Random intercepts account for within‐subject correlation. For within‐group CID, mean changes in PROM are estimated across anchor‐defined categories. Minimal, moderate and marked improvements can be identified based on anchor values (e.g., PGI‐C = 5 for minimal improvement). For between‐group CID, differences in means between anchor categories (e.g., PGI‐C = 4 [no change] vs. PGI‐C = 5 [minimal improvement]) are interpreted as a minimal CID threshold.
(6) *Visualisation and output*: The macro generates forest plots of estimated PROM changes (with 95% confidence intervals [CIs]) stratified by anchor categories. The reference level (e.g., PGI‐C = 4) is highlighted to aid interpretation of minimal change thresholds and to assess potential placebo effects or disease regression. Categorical differences (e.g., PGI‐C = 4 vs. PGI‐C = 5) provide visual estimates of between‐group CID thresholds.

### The ESTICID R Shiny application

**FIGURE 1:**
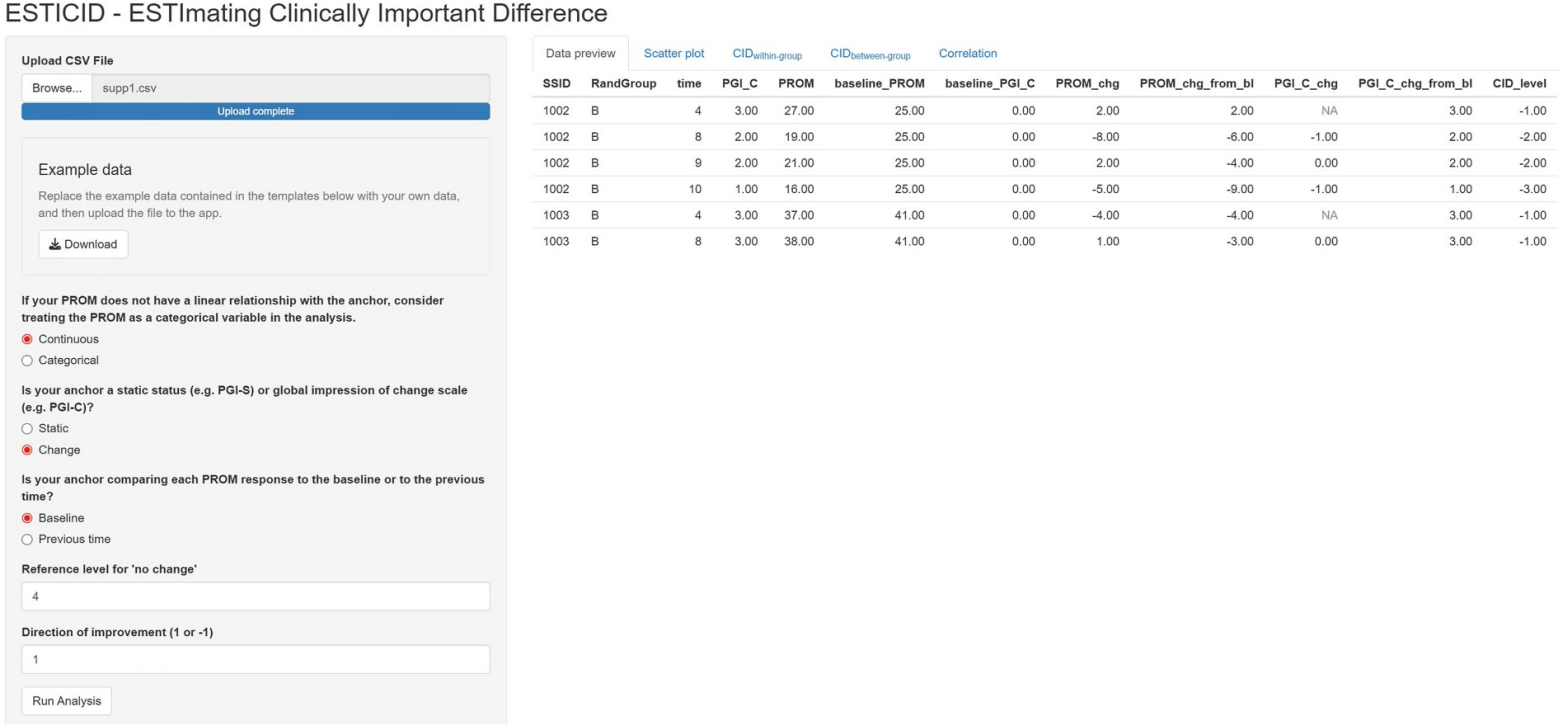
Interface of the estimating clinically important differences R Shiny application.

The ESTICID R Shiny application is freely accessible via a web browser and is intended to support users who prefer a graphical interface without coding (https://kuanliao.shinyapps.io/ESTICID/). The application includes a data upload panel, parameter setting options and the output panel. Users can upload data in the same format as the example data provided in Supplemental File 1. Five parameters are required to be prespecified: (1) the anchor type (continuous vs. categorical), (2) whether the anchor reflects a static status or a change score, (3) whether each PROM response is compared with baseline or with the immediately preceding assessment, (4) the reference category corresponding to ‘no change’ in the anchor variable and (5) the direction of improvement (ascending vs. descending scores). The output section includes a data preview tag presenting the first six rows of the uploaded data, a scatter plot tab visualising the correlation between the anchor and the PROM, as well as CID_within‐group_, CID_between‐group_ and associated graphical summaries. Figure 1 shows the interface of the ESTICID R Shiny application.

### Implementation of the %ESTICID SAS macro

The %ESTICID macro is designed for SAS users to estimate CIDs using an anchor‐based approach. It requires a single input dataset in long format, with one row per patient per time point. The syntax is %ESTICID (data=, id=, time=, anchor=, prom=, categoric_anchor=, covstruct=, reference=, improve_dir=). The main macro parameters are as follows: (1) data: name of the input SAS dataset in long format; (2) id: unique subject identifier variable (one per patient); (3) time: time or visit variable (numeric or date), indicating observation order. The earliest time value is assumed to represent baseline; (4) anchor: anchor variable used for CID estimation (e.g., PGI‐C). The anchor can be numeric or character, but levels must be logically ordered (e.g., from ‘much worse’ to ‘much better’); (5) prom: patient‐reported outcome measure (numeric score), collected at baseline and follow‐up(s); (6) categoric_anchor: specifies whether the anchor should be treated as a categorical (Y) or continuous (N) variable in the model; (7) covstruct: covariance structure used in the REPEATED statement of the mixed‐effects model; (8) reference: defines the anchor category corresponding to ‘no change’ and is used to standardise anchor levels for CID estimation; and (9) improve_dir: direction for improvement, −1 (minimise metric) and 1 (maximise metric).

### Examples

We illustrate ESTICID using data from a multicenter, randomised clinical trial.^21^ The trial enrolled male patients diagnosed with chronic prostatitis/chronic pelvic pain syndrome (CP/CPPS) and aimed to evaluate the clinical effectiveness and safety of acupuncture compared to sham acupuncture. A total of 414 eligible participants had experienced pelvic or prostate‐region pain for at least 3 out of the previous 6 months, without other identifiable urinary tract pathology. The dataset structure corresponds to long‐format data (Supplemental File 1), where the anchor (PGI‐C) was assessed only post‐treatment and no baseline anchor values were available. The dataset must include at least one baseline and one follow‐up observation per subject. The ESTICID automatically identifies the baseline as the earliest time point for each subject. PROM changes from baseline are computed for all non‐baseline time points. The mixed‐effects model uses all available follow‐up observations under the specified model and a missing‐at‐random (MAR) assumption. The ESTICID supports multiple follow‐up time points and can accommodate unbalanced or incomplete longitudinal data. This structure ensures compatibility with standard clinical trial or observational datasets and allows flexible modelling of both CID_within‐group_ and CID_between‐group_ based on the anchor‐PROM relationship.

**FIGURE 2:**
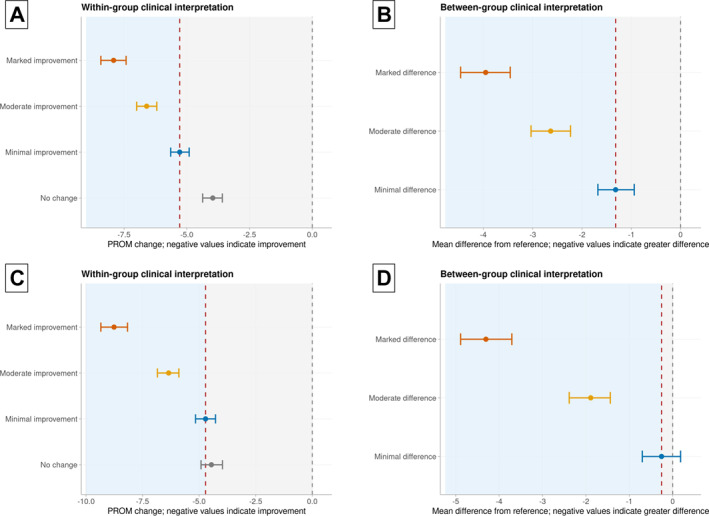
Estimated CID_within‐group_ and CID_between‐group_ for minimal, moderate, and marked categories using estimating clinically important differences. (A) and (C) show within‐group patient‐reported outcome measure changes across anchor‐defined categories. (B) and (D) show between‐group mean differences relative to the reference category. (A) and (B) present results from the continuous‐anchor specification, and (C) and (D) present results from the categorical‐anchor specification. Points represent model‐based estimates, and horizontal bars represent 95% confidence intervals. Negative values indicate greater improvement. The red dashed line indicates the minimal clinically important threshold, and the grey dashed line indicates no change or no between‐group difference.

The primary PROM was the National Institutes of Health Chronic Prostatitis Symptom Index total score, measured at baseline and multiple follow‐up visits. The anchor, PGI‐C, was treated as a categorical variable. To estimate the CIDs for both between‐group and within‐group comparisons, we used the ESTICID to generate several diagnostic and visualisation outputs. Before estimating CIDs, we assessed the validity of the anchor by quantifying its association with PROM scores. Specifically, we calculated the correlation between the anchor and the PROM at corresponding post‐baseline visits. Consistent with commonly recommended criteria for anchor‐based CID studies, we prespecified that the anchor should demonstrate at least a moderate association with the PROM (e.g., an absolute correlation coefficient ≥ 0.30). Otherwise, we recommend using an alternative anchor or treating the anchor categorically and interpreting estimates cautiously. Then, forest plots (figure 2A, C) summarised the estimated PROM changes across PGI‐C categories, along with 95% CIs. This graphical output facilitates interpretation of CID_within‐group_ estimation, enabling clinicians and guideline developers to judge whether observed changes reflect minimal, moderate or marked clinical improvement. Based on the reference anchor, ESTICID also provides CID_between‐group_ estimation (figure 2B, D). Outputs from the R Shiny application and SAS macro are equivalent. The SAS and R code are provided as a supplemental file to support reproducibility and transparency (Supplemental File 2).

We applied the ESTICID to evaluate the robustness of CID estimation under varying degrees of missing data. Starting with a complete longitudinal dataset (*n* = 414), we simulated missingness under the MAR assumption, with 10%, 20%, 30% and 40% of follow‐up observations based on baseline values (patients with worse baseline severity are more likely to drop out) and clinical outcomes. This approach mimics the patterns of attrition and nonresponse commonly encountered in real‐world clinical studies. The results demonstrated high stability of the CID estimates across all levels of missingness (Supplemental File 3). Across all scenarios, the estimated CIDs and their 95% CIs remained stable, with only minimal deviations observed at higher levels of missingness. These findings support the robustness of the mixed‐effects modelling framework implemented in ESTICID for unbalanced longitudinal data commonly encountered in clinical research.

## DISCUSSION

In this study, we developed ESTICID, a cross‐platform toolkit for estimating and visualising CIDs in longitudinal PROM data using both R Shiny and SAS. Rather than serving only as a software implementation, ESTICID is intended to support a clinically oriented framework for PROM interpretation by distinguishing CID_within‐group_ from CID_between‐group_ and by linking these estimands to anchor‐based mixed‐effects modelling. This distinction is particularly relevant in psychiatry and related fields, where symptom scales and PROMs are widely used but the clinical meaning of observed changes is often difficult to interpret. The relevance of this framework to psychiatry deserves particular emphasis. Psychiatric clinical trials and observational studies frequently rely on PROMs to evaluate treatment benefit, yet statistically significant findings do not necessarily indicate clinically meaningful change. A transparent and accessible approach to CID estimation may therefore help psychiatric researchers, clinicians and guideline developers move from numerical score changes to clinically interpretable judgements regarding benefit, responder definition and decision thresholds.

One major strength of this approach is its patient‐centred nature. The anchor variable, such as a global impression of change in symptoms (e.g., PGI‐C), reflects the patient's subjective perception of improvement or worsening, thereby grounding the definition of clinically meaningful change in real‐world relevance. Anchor‐based methods are widely regarded as more interpretable than purely statistical or distribution‐based approaches, because they reflect what patients themselves consider a meaningful difference. Another key advantage lies in the use of mixed‐effects models. By employing an LMM, the macro accounts for the correlation of repeated measures within individuals and accommodates incomplete or unbalanced longitudinal data. The inclusion of least square means allows estimation of adjusted PROM changes across anchor categories, complete with CIs, which are essential for interpretation in clinical research. The macro also enhances communication of findings through graphical output. The forest plot it generates, showing mean PROM change by anchor level along with 95% CIs, provides an intuitive and accessible summary of the magnitude of change. This format has proven effective in supporting both clinician judgement and shared decision‐making.

Compared with distribution‐based or ROC‐based approaches, ESTICID emphasises anchor‐based interpretability whilst accommodating the practical complexity of longitudinal clinical data.^20^ ROC‐based methods can be useful for identifying classification thresholds, but they often require dichotomisation of anchors or outcomes and are less flexible when anchors are ordinal, repeated measures are available or follow‐up data are incomplete.^22^ In contrast, the mixed‐effects framework used in ESTICID allows the full longitudinal structure of the data to be retained, supports both continuous and categorical anchors and yields estimates that are directly interpretable in relation to patient‐perceived change. To improve transparency and reproducibility of CID estimates, we recommend reporting the data source, participants, time frame, type of anchor, estimation methods and sample size.

Despite these strengths, several considerations should guide the application of ESTICID beyond the illustrative dataset. ESTICID is most appropriate for longitudinal clinical studies or trials in which PROMs are repeatedly measured, an external anchor is clinically interpretable and the anchor demonstrates an adequate association with PROM change. If the anchor is weakly correlated with PROM change, the resulting CID estimates may lack clinical validity. In such cases, alternative anchors, sensitivity analyses or a mixed‐method approach should be considered. The toolkit is intended to estimate group‐level CID values, including CID_within‐group_ and CID_between‐group_, rather than to provide definitive individual‐level thresholds for determining whether a specific patient has improved. When anchor categories contain very small numbers of participants, estimates for minimal, moderate or marked improvement may be unstable and should be interpreted cautiously. CID estimates should also be interpreted as context‐specific rather than fixed measurement constants. They may vary according to population, baseline severity, follow‐up duration, treatment, language or cultural setting, PROM instrument and anchor definition. Therefore, CID values estimated in one dataset should not be generalised uncritically to populations or settings that differ substantially from the source study. These considerations reinforce that ESTICID should be viewed as a structured framework for estimating and communicating anchor‐based CID values, not as an automatic substitute for clinical judgement. We recommend that future applications report the study population, PROM, anchor, anchor‐PROM correlation, follow‐up time frame, sample size within each anchor category, missing‐data pattern, estimation method and whether the estimate refers to CID_within‐group_ or CID_between‐group_. Such reporting will help users judge whether a CID estimate is transportable to their own clinical or research context.

In summary, ESTICID provides a practical, interpretable and accessible framework for anchor‐based CID estimation in longitudinal PROM research. By integrating conceptual clarity, graphical output and cross‐platform implementation, it may improve the consistency and clinical interpretability of PROM reporting in psychiatry and other clinical fields.

## FUNDING

This study was funded by the National Natural Science Foundation of China (82304973), the International Postdoctoral Exchange Fellowship Program (YJ20220238), and the China Postdoctoral Science Foundation (2023TQ0018).

## CONFLICT OF INTEREST STATEMENT

The authors declare no conflicts of interest.

## PATIENT CONSENT FOR PUBLICATION

Not applicable.

## ETHICS STATEMENT

Not applicable.

## Supporting information

Supporting Information S1

Supporting Information S2

Supporting Information S3

## Data Availability

The shared dataset used for illustration has been provided in Supplementary File 1.

## References

[1] Cella D , Riley W , Stone A , Rothrock N , Reeve B , Yount S , et al. The Patient‐Reported Outcomes Measurement Information System (PROMIS) developed and tested its first wave of adult self‐reported health outcome item banks: 2005–2008. J Clin Epidemiol. 2010;63(11):1179–1194. 10.1016/j.jclinepi.2010.04.011 20685078 PMC2965562

[2] Evans JP , Smith A , Gibbons C , Alonso J , Valderas JM . The National Institutes of Health Patient‐Reported Outcomes Measurement Information System (PROMIS): a view from the UK. Patient Relat Outcome Meas. 2018;9:345–352. 10.2147/prom.s141378 30498382 PMC6207259

[3] Qin Z , Zhu Y , Shi DD , Chen R , Li S , Wu J . The gap between statistical and clinical significance: time to pay attention to clinical relevance in patient‐reported outcome measures of insomnia. BMC Med Res Methodol. 2024;24(1):177. 10.1186/s12874-024-02297-0 39118002 PMC11308508

[4] Amrhein V , Greenland S , McShane B . Scientists rise up against statistical significance. Nature. 2019;567(7748):305–307. 10.1038/d41586-019-00857-9 30894741

[5] Desai N , Albrecht E . Minimal clinically important difference: bridging the gap between statistical significance and clinical meaningfulness. J Clin Anesth. 2024;94:111366. 10.1016/j.jclinane.2023.111366 38244304

[6] Guyatt G , Walter S , Norman G . Measuring change over time: assessing the usefulness of evaluative instruments. J Chron Dis. 1987;40(2):171–178. 10.1016/0021-9681(87)90069-5 3818871

[7] King MT . A point of minimal important difference (MID): a critique of terminology and methods. Expert Rev Pharmacoecon Outcomes Res. 2011;11(2):171–184. 10.1586/erp.11.9 21476819

[8] Wu J , Yao L , Liao K , Yorke J , Yu Y , Liu Z , et al. Estimating minimal, moderate, and marked clinically important differences for patient reported outcome measure in randomised controlled trial. CMJ. 2026;139(10):1538–1544. 10.1097/CM9.0000000000004084 PMC13186122 41914019

[9] Morgano GP , Wiercioch W , Piovani D , Neumann I , Nieuwlaat R , Piggott T , et al. Defining decision thresholds for judgments on health benefits and harms using the grading of recommendations assessment, development, and evaluation (GRADE) evidence to decision (EtD) frameworks: a randomized methodological study (GRADE‐THRESHOLD). J Clin Epidemiol. 2025;179:111639. 10.1016/j.jclinepi.2024.111639 39662641

[10] Alonso‐Coello P , Schunemann HJ , Moberg J , Brignardello‐Petersen R , Akl EA , Davoli M , et al. GRADE Evidence to Decision (EtD) frameworks: a systematic and transparent approach to making well informed healthcare choices. 1: introduction. BMJ. 2016;353:i2016. 10.1136/bmj.i2016 27353417

[11] Puhan MA , Singh S , Weiss CO , Varadhan R , Boyd CM . A framework for organizing and selecting quantitative approaches for benefit‐harm assessment. BMC Med Res Methodol. 2012;12(1):173. 10.1186/1471-2288-12-173 23163976 PMC3572426

[12] Stephens‐Shields AJ , Lai HH , Landis JR , Kreder K , Rodriguez LV , Naliboff BD , et al. Clinically important differences for pain and urinary symptoms in urological chronic pelvic pain syndrome: a MAPP network study. J Urol. 2023;209(6):1132–1140. 10.1097/ju.0000000000003394 36848118 PMC11062515

[13] Terwee CB , Peipert JD , Chapman R , Lai JS , Terluin B , Cella D , et al. Minimal important change (MIC): a conceptual clarification and systematic review of MIC estimates of PROMIS measures. Qual Life Res. 2021;30(10):2729–2754. 10.1007/s11136-021-02925-y 34247326 PMC8481206

[14] Neely JG , Karni RJ , Engel SH , Fraley PL , Nussenbaum B , Paniello RC . Practical guides to understanding sample size and minimal clinically important difference (MCID). Otolaryngol Head Neck Surg. 2007;136(1):14–18. 10.1016/j.otohns.2006.11.001 17210326

[15] Mauri L , D'Agostino RB Sr . Challenges in the design and interpretation of noninferiority trials. N Engl J Med. 2017;377(14):1357–1367. 10.1056/nejmra1510063 28976859

[16] Dekker J , de Boer M , Ostelo R . Minimal important change and difference in health outcome: an overview of approaches, concepts, and methods. Osteoarthr Cartil. 2024;32(1):8–17. 10.1016/j.joca.2023.09.002 37714259

[17] Guyatt GH , Osoba D , Wu AW , Wyrwich KW , Norman GR , Clinical Significance Consensus Meeting Group . Methods to explain the clinical significance of health status measures. Mayo Clin Proc. 2002;77(4):371–383. 10.4065/77.4.371 11936935

[18] Baker A , Simon N , Keshaviah A , Farabaugh A , Deckersbach T , Worthington JJ , et al. Anxiety Symptoms Questionnaire (ASQ): development and validation. Gen Psychiatr. 2019;32(6):e100144. 10.1136/gpsych-2019-100144 31922090 PMC6936972

[19] Gu X , Su YA , Lin J , Chen X , Bushnell DM , Fu D , et al. Quantitative scale validation of the Dimensional Anhedonia Rating Scale in the treatment of Chinese patients with major depressive disorder. Gen Psychiatr. 2025;38(2):e101789. 10.1136/gpsych-2024-101789 40191017 PMC11969601

[20] Kersey J , Baum CM , Hammel J , Terhorst L , McCue M , Skidmore ER . Cut points and sensitivity to change of the Enfranchisement scale of the Community Participation Indicators in adults with traumatic brain injury. PM & R. 2023;15(2):176–183. 10.1002/pmrj.12743 34865309

[21] Sun Y , Liu Y , Liu B , Zhou K , Yue Z , Zhang W , et al. Efficacy of acupuncture for chronic prostatitis/chronic pelvic pain syndrome: a randomized trial. Ann Intern Med. 2021;174(10):1357–1366. 10.7326/m21-1814 34399062

[22] Twisk J , de Boer M , de Vente W , Heymans M . Multiple imputation of missing values was not necessary before performing a longitudinal mixed‐model analysis. J Clin Epidemiol. 2013;66(9):1022–1028. 10.1016/j.jclinepi.2013.03.017 23790725

