# Supplementary material for: Enhancing clinical interpretation of patient‐reported outcomes: A cross‐platform toolkit for clinically important difference estimation using R Shiny and statistical analysis system
Source: Gen Psychiatr. 2026 Aug 13;39(4):e70041. doi: 10.1002/gps3.70041 (PMC13473175; doi:10.1002/gps3.70041)
Supplement: Supplementary file 1 [file GPS3-39-e70041-s001.pdf]

Sensitivity analysis under Missing At Random (MAR) mechanism

Missing (%)	Label	Estimates	95% Confidence Interval	
10	No change	-3.6715	-4.1016	-3.2414
10	Minimal CID within-group	-5.1129	-5.4844	-4.7415
10	Moderate CID within-group	-6.5544	-6.9468	-6.1620
10	Marked CID within-group	-7.9958	-8.4786	-7.5130
10	Minimal CID between-group	-1.4414	-1.6193	-1.2636
10	Moderate CID between-group	-2.8829	-3.2386	-2.5271
10	Marked CID between-group	-4.3243	-4.8580	-3.7907
20	No change	-3.3344	-3.7674	-2.9014
20	Minimal CID within-group	-4.9134	-5.2765	-4.5503
20	Moderate CID within-group	-6.4924	-6.8760	-6.1087
20	Marked CID within-group	-8.0714	-8.5547	-7.5881
20	Minimal CID between-group	-1.5790	-1.7677	-1.3902
20	Moderate CID between-group	-3.1580	-3.5355	-2.7805
20	Marked CID between-group	-4.7370	-5.3032	-4.1707
30	No change	-3.0211	-3.4715	-2.5706
30	Minimal CID within-group	-4.7448	-5.1087	-4.3808
30	Moderate CID within-group	-6.4684	-6.8550	-6.0819
30	Marked CID within-group	-8.1921	-8.6960	-7.6882
30	Minimal CID between-group	-1.7237	-1.9331	-1.5143
30	Moderate CID between-group	-3.4474	-3.8662	-3.0286
30	Marked CID between-group	-5.1711	-5.7992	-4.5429
40	No change	-2.6549	-3.1289	-2.1808
40	Minimal CID within-group	-4.5007	-4.8644	-4.1370
40	Moderate CID within-group	-6.3465	-6.7328	-5.9603
40	Marked CID within-group	-8.1924	-8.7173	-7.6674
40	Minimal CID between-group	-1.8458	-2.0800	-1.6117
40	Moderate CID between-group	-3.6917	-4.1599	-3.2234
40	Marked CID between-group	-5.5375	-6.2399	-4.8351

Note: sensitivity analysis using a MAR mechanism, where the probability of PROM missingness depended on observed covariates (baseline and time). Specifically, for each post-baseline observation we generated a missingness indicator using a logistic model with baseline PROM and follow-up time as predictors, and tuned the intercept to obtain approximately 10%, 20%, 30%, and 40% overall missingness, respectively.
