# Supplementary material for: Enhancing clinical interpretation of patient‐reported outcomes: A cross‐platform toolkit for clinically important difference estimation using R Shiny and statistical analysis system
Source: Gen Psychiatr. 2026 Aug 13;39(4):e70041. doi: 10.1002/gps3.70041 (PMC13473175; doi:10.1002/gps3.70041)
Supplement: Supplementary file 2 [file GPS3-39-e70041-s003.pdf]

ESTICID R code

```
#required packages
packages <- c("shiny", "dplyr", "lme4", "ggplot2", "boot")

#Identify which packages are not already installed
new_packages <- packages[!(packages %in% installed.packages()[,"Package"])]

# Install the missing packages
if(length(new_packages)) {
  install.packages(new_packages)
}

#Library the packages
lapply(packages, library, character.only = TRUE)


########## UI ##########
ui <- fluidPage(
  titlePanel("CIDER - Clinically Important Difference Estimation and Rating using anchor-based approach"),

  sidebarLayout(
    sidebarPanel(
      fileInput("upload", "Upload CSV File", accept = ".csv"),

      radioButtons(
        inputId = "PROM_type",
        label = "If your PROM does not have a linear relationship with the anchor, consider treating the PROM as a categorical variable in the analysis.",
        choices = c("Continuous" = "continuous", "Categorical" = "categorical")
      ),

      radioButtons(
        inputId = "Anchor_type",
        label = "Is your anchor a static status (e.g. PGI-S) or global impression of change scale (e.g. PGI-C)?",
        choices = c("Static" = "static", "Change" = "change")
      ),

      radioButtons(
        inputId = "Comparison",
        label = "Is your anchor comparing each PROM response to the baseline or to the previous time?",
```

```
      choices = c("Baseline" = "baseline", "Previous time" = "last")
    ),

    numericInput("reference", "Reference level for 'no change'", value = 4, min = -999, max =
999, step = 1),

    numericInput("improve_dir", "Direction of improvement (1 or -1)", value = 1, min = -1,
max = 1, step = 2),

    actionButton("run", "Run Analysis")
  ),

  mainPanel(
    tabsetPanel(
      tabPanel("Data Preview", tableOutput("headdf")),
      tabPanel("Scatter Plot", plotOutput("scatter_plot")),
      tabPanel("CID Within-group",
               fluidRow(
                 column(6, plotOutput("forest_plot_within")),
                 column(6, tableOutput("ci_table_within"))
               )),
      tabPanel("CID Between-Group",
               fluidRow(
                 column(6, plotOutput("forest_plot_between")),
                 column(6, tableOutput("ci_table_between"))
               )),
      tabPanel("Correlation", tableOutput("corr_table"))
    )
  )
 )
)

########## Server ##########
server <- function(input, output) {

 raw_data <- reactive({
  req(input$upload)
  read.csv(input$upload$datapath)
 })

 processed_data <- eventReactive(input$run, {
  df <- raw_data()

  # Basic required columns
```

```r
if (!all(c("SSID", "time", "PROM") %in% names(df))) {
  stop("Required columns: SSID, time, PROM.")
}

df <- df %>%
  mutate(
    SSID = as.factor(SSID),
    time = as.integer(time),
    PROM = as.numeric(PROM)
  )

if (input$Anchor_type == "static") {
  if (!"PGI_S" %in% names(df)) stop("Column 'PGI_S' not found in your data.")
  df <- df %>%
    mutate(PGI_S = as.numeric(PGI_S)) %>%
    arrange(SSID, time) %>%
    group_by(SSID) %>%
    mutate(
      baseline_PROM = coalesce(first(PROM), 0),
      baseline_PGI_S = coalesce(first(PGI_S), 0),
      PROM_chg = PROM - lag(PROM),
      PROM_chg_from_bl = PROM - baseline_PROM,
      PGI_S_chg = PGI_S - lag(PGI_S),
      PGI_S_chg_from_bl = PGI_S - baseline_PGI_S,
      CID_level = (PGI_S_chg_from_bl - input$reference) * input$improve_dir
    ) %>%
    ungroup()

} else if (input$Anchor_type == "change") {
  if (!"PGI_C" %in% names(df)) stop("Column 'PGI_C' not found in your data.")
  df <- df %>%
    mutate(PGI_C = as.numeric(PGI_C)) %>%
    arrange(SSID, time) %>%
    group_by(SSID) %>%
    mutate(
      baseline_PROM = coalesce(first(PROM), 0),
      baseline_PGI_C = coalesce(first(PGI_C), 0),
      PROM_chg = PROM - lag(PROM),
      PROM_chg_from_bl = PROM - baseline_PROM,
      PGI_C_chg = PGI_C - lag(PGI_C),
      PGI_C_chg_from_bl = PGI_C - baseline_PGI_C,
      CID_level = (PGI_C_chg_from_bl - input$reference) * input$improve_dir
    ) %>%
    ungroup()
```

```
    } else {
      stop("Unknown anchor type.")
    }

    # Exclude baseline rows (where lag is NA)
    df <- df %>% filter(!is.na(PROM_chg))

    if (nrow(df) == 0) {
      stop("No follow-up observations found after baseline exclusion.")
    }

    df
  })

  model_fit <- reactive({
    req(processed_data(), input$PROM_type, input$Anchor_type, input$Comparison)
    df <- processed_data()

    if (input$PROM_type == "continuous" & input$Anchor_type == "change" &
input$Comparison == "baseline") {
      lmer(PROM_chg_from_bl ~ PGI_C + (1 | SSID), data = df)
    } else if (input$PROM_type == "continuous" & input$Anchor_type == "change" &
input$Comparison == "last") {
      lmer(PROM_chg ~ PGI_C + (1 | SSID), data = df)
    } else if (input$PROM_type == "categorical" & input$Anchor_type == "change" &
input$Comparison == "baseline") {
      lmer(PROM_chg_from_bl ~ as.factor(PGI_C) + (1 | SSID), data = df)
    } else if (input$PROM_type == "categorical" & input$Anchor_type == "change" &
input$Comparison == "last") {
      lmer(PROM_chg ~ as.factor(PGI_C) + (1 | SSID), data = df)
    } else if (input$PROM_type == "continuous" & input$Anchor_type == "static" &
input$Comparison == "baseline") {
      lmer(PROM_chg_from_bl ~ PGI_S_chg_from_bl + (1 | SSID), data = df)
    } else if (input$PROM_type == "continuous" & input$Anchor_type == "static" &
input$Comparison == "last") {
      lmer(PROM_chg ~ PGI_S_chg + (1 | SSID), data = df)
    } else if (input$PROM_type == "categorical" & input$Anchor_type == "static" &
input$Comparison == "baseline") {
      lmer(PROM_chg_from_bl ~ as.factor(PGI_S_chg_from_bl) + (1 | SSID), data = df)
    } else if (input$PROM_type == "categorical" & input$Anchor_type == "static" &
input$Comparison == "last") {
      lmer(PROM_chg ~ as.factor(PGI_S_chg) + (1 | SSID), data = df)
    } else {
```

```r
      NULL
    }
  })

  output$headdf <- renderTable({
    req(processed_data())
    head(processed_data())
  })

  output$scatter_plot <- renderPlot({
    req(processed_data(), input$PROM_type, input$Anchor_type, input$Comparison)
    df <- processed_data()

    if (input$Anchor_type == "change" & input$Comparison == "baseline") {
      ggplot(df, aes(x = PGI_C, y = PROM_chg_from_bl)) +
        geom_point(color = "grey30") +
        geom_smooth(method = "lm", se = TRUE, color = "red") +
        labs(x = "Patient Global Impression of Change (PGI-C)",
             y = "Change in PROM from Baseline")
    } else if (input$Anchor_type == "change" & input$Comparison == "last") {
      ggplot(df, aes(x = PGI_C, y = PROM_chg)) +
        geom_point(color = "grey30") +
        geom_smooth(method = "lm", se = TRUE, color = "red") +
        labs(x = "Patient Global Impression of Change (PGI-C)",
             y = "Change in PROM from last response")
    } else if (input$Anchor_type == "static" & input$Comparison == "baseline") {
      ggplot(df, aes(x = PGI_S_chg_from_bl, y = PROM_chg_from_bl)) +
        geom_point(color = "grey30") +
        geom_smooth(method = "lm", se = TRUE, color = "red") +
        labs(x = "Patient Global Impression of Severity (PGI-S)",
             y = "Change in PROM from Baseline")
    } else {
      ggplot(df, aes(x = PGI_S_chg, y = PROM_chg)) +
        geom_point(color = "grey30") +
        geom_smooth(method = "lm", se = TRUE, color = "red") +
        labs(x = "Patient Global Impression of Severity (PGI-S)",
             y = "Change in PROM from last response")
    }
  })

  output$corr_table <- renderTable({
    req(processed_data())
    df <- processed_data()
    cor_data <- data.frame(
```

```
      CID_level = df$CID_level,
      PROM_change = if (input$Comparison == "baseline") df$PROM_chg_from_bl else
df$PROM_chg
    )
    cor_val <- cor(cor_data$CID_level, cor_data$PROM_change, use = "pairwise.complete.obs")
    data.frame(
      Variable = "CID_level vs PROM_change",
      Correlation = round(cor_val, 4)
    )
  }, striped = TRUE)

  output$forest_plot_within <- renderPlot({
    req(input$run, model_fit(), input$PROM_type, input$Anchor_type)
    model <- model_fit()

    new_data <- if (input$PROM_type == "continuous" & input$Anchor_type == "change") {
      data.frame(PGI_C = 1:4, SSID = NA)
    } else if (input$PROM_type == "categorical" & input$Anchor_type == "change") {
      data.frame(PGI_C = as.factor(1:4), SSID = NA)
    } else if (input$PROM_type == "continuous" & input$Anchor_type == "static" &
input$Comparison == "last") {
      data.frame(PGI_S_chg = 1:4, SSID = NA)
    } else if (input$PROM_type == "continuous" & input$Anchor_type == "static" &
input$Comparison == "baseline") {
      data.frame(PGI_S_chg_from_bl = 1:4, SSID = NA)
    } else if (input$PROM_type == "categorical" & input$Anchor_type == "static" &
input$Comparison == "last") {
      data.frame(PGI_S_chg = as.factor(1:4), SSID = NA)
    } else if (input$PROM_type == "categorical" & input$Anchor_type == "static" &
input$Comparison == "baseline") {
      data.frame(PGI_S_chg_from_bl = as.factor(1:4), SSID = NA)
    }

    boot_fun <- function(fit) predict(fit, newdata = new_data, re.form = NA)
    set.seed(123)
    boot_res <- bootMer(model, boot_fun, nsim = 1000)
    cis <- apply(boot_res$t, 2, quantile, probs = c(0.025, 0.975))

    new_data$lower_ci <- cis[1, ]
    new_data$upper_ci <- cis[2, ]
    new_data$predicted <- predict(model, newdata = new_data, re.form = NA)

    plot_data <- if (input$Anchor_type == "change") {
      new_data %>% mutate(PGI = PGI_C)
```

```r
    } else if (input$Anchor_type == "static" & input$Comparison == "baseline") {
      new_data %>% mutate(PGI = PGI_S_chg_from_bl)
    } else {
      new_data %>% mutate(PGI = PGI_S_chg)
    }

    ggplot(plot_data, aes(x = predicted, y = as.factor(PGI))) +
      geom_point(color = "grey10", size = 2) +
      geom_errorbarh(aes(xmin = lower_ci, xmax = upper_ci), height = 0.2, color = "grey30") +
      geom_vline(xintercept = 0, linetype = "dashed", color = "grey50") +
      labs(
        x = "Predicted PROM Change (Within-Group)",
        y = ifelse(input$Anchor_type == "change", "PGI-C", "PGI-S"),
        title = paste("Within-Group Forest Plot (", input$PROM_type, ")", sep = "")
      ) +
      theme_bw()
  })

  output$forest_plot_between <- renderPlot({
    req(input$run, model_fit(), input$PROM_type, input$Anchor_type)
    model <- model_fit()

    new_data <- if (input$PROM_type == "continuous" & input$Anchor_type == "change") {
      data.frame(PGI_C = 1:4, SSID = NA)
    } else if (input$PROM_type == "categorical" & input$Anchor_type == "change") {
      data.frame(PGI_C = as.factor(1:4), SSID = NA)
    } else if (input$PROM_type == "continuous" & input$Anchor_type == "static" &
input$Comparison == "last") {
      data.frame(PGI_S_chg = 1:4, SSID = NA)
    } else if (input$PROM_type == "continuous" & input$Anchor_type == "static" &
input$Comparison == "baseline") {
      data.frame(PGI_S_chg_from_bl = 1:4, SSID = NA)
    } else if (input$PROM_type == "categorical" & input$Anchor_type == "static" &
input$Comparison == "last") {
      data.frame(PGI_S_chg = as.factor(1:4), SSID = NA)
    } else if (input$PROM_type == "categorical" & input$Anchor_type == "static" &
input$Comparison == "baseline") {
      data.frame(PGI_S_chg_from_bl = as.factor(1:4), SSID = NA)
    }

    boot_fun <- function(fit) predict(fit, newdata = new_data, re.form = NA)
    set.seed(123)
    boot_res <- bootMer(model, boot_fun, nsim = 1000)
    cis <- apply(boot_res$t, 2, quantile, probs = c(0.025, 0.975))
```

```
    new_data$lower_ci <- cis[1, ]
    new_data$upper_ci <- cis[2, ]
    new_data$predicted <- predict(model, newdata = new_data, re.form = NA)

    ref_idx <- which(if (input$Anchor_type == "change") new_data$PGI_C == input$reference
else {
      if (input$Comparison == "baseline") new_data$PGI_S_chg_from_bl == input$reference
else new_data$PGI_S_chg == input$reference
    })
    ref_val <- new_data$predicted[ref_idx][1]

    plot_data <- if (input$Anchor_type == "change") {
      new_data %>% mutate(PGI = PGI_C)
    } else if (input$Anchor_type == "static" & input$Comparison == "baseline") {
      new_data %>% mutate(PGI = PGI_S_chg_from_bl)
    } else {
      new_data %>% mutate(PGI = PGI_S_chg)
    }

    plot_data <- plot_data %>%
      mutate(diff_from_ref = predicted - ref_val,
             lower_diff = lower_ci - ref_val,
             upper_diff = upper_ci - ref_val) %>%
      filter(as.numeric(PGI) != input$reference)

    ggplot(plot_data, aes(x = diff_from_ref, y = as.factor(PGI))) +
      geom_point(color = "grey10", size = 2) +
      geom_errorbarh(aes(xmin = lower_diff, xmax = upper_diff), height = 0.2, color = "grey30")
+
      geom_vline(xintercept = 0, linetype = "dashed", color = "grey50") +
      labs(
        x = "Mean Difference from Reference (Between-Group)",
        y = ifelse(input$Anchor_type == "change", "PGI-C", "PGI-S"),
        title = paste("Between-Group Forest Plot (", input$PROM_type, ")", sep = "")
      ) +
      theme_bw()
  })

  output$ci_table_within <- renderTable({
    req(model_fit())
    model <- model_fit()

    new_data <- if (input$PROM_type == "continuous" & input$Anchor_type == "change") {
```

```
        data.frame(PGI_C = 1:4, SSID = NA)
      } else if (input$PROM_type == "categorical" & input$Anchor_type == "change") {
        data.frame(PGI_C = as.factor(1:4), SSID = NA)
      } else if (input$PROM_type == "continuous" & input$Anchor_type == "static" &
input$Comparison == "last") {
        data.frame(PGI_S_chg = 1:4, SSID = NA)
      } else if (input$PROM_type == "continuous" & input$Anchor_type == "static" &
input$Comparison == "baseline") {
        data.frame(PGI_S_chg_from_bl = 1:4, SSID = NA)
      } else if (input$PROM_type == "categorical" & input$Anchor_type == "static" &
input$Comparison == "last") {
        data.frame(PGI_S_chg = as.factor(1:4), SSID = NA)
      } else if (input$PROM_type == "categorical" & input$Anchor_type == "static" &
input$Comparison == "baseline") {
        data.frame(PGI_S_chg_from_bl = as.factor(1:4), SSID = NA)
      }

      boot_fun <- function(fit) predict(fit, newdata = new_data, re.form = NA)
      set.seed(123)
      boot_res <- bootMer(model, boot_fun, nsim = 1000)
      cis <- apply(boot_res$t, 2, quantile, probs = c(0.025, 0.975))

      new_data$lower_ci <- cis[1, ]
      new_data$upper_ci <- cis[2, ]
      new_data$predicted <- predict(model, newdata = new_data, re.form = NA)

      if (input$Anchor_type == "change") {
        new_data %>%
          mutate(Expected_PROM = predicted) %>%
          arrange(desc(as.numeric(PGI_C))) %>%
          select(PGI_C, Expected_PROM, lower_ci, upper_ci)
      } else if (input$Anchor_type == "static" & input$Comparison == "baseline") {
        new_data %>%
          mutate(PGI_S = PGI_S_chg_from_bl, Expected_PROM = predicted) %>%
          arrange(desc(as.numeric(PGI_S))) %>%
          select(PGI_S, Expected_PROM, lower_ci, upper_ci)
      } else {
        new_data %>%
          mutate(PGI_S = PGI_S_chg, Expected_PROM = predicted) %>%
          arrange(desc(as.numeric(PGI_S))) %>%
          select(PGI_S, Expected_PROM, lower_ci, upper_ci)
      }
    }, striped = TRUE)
```

```
output$ci_table_between <- renderTable({
    req(model_fit(), input$reference)
    model <- model_fit()

    new_data <- if (input$PROM_type == "continuous" & input$Anchor_type == "change") {
        data.frame(PGI_C = 1:4, SSID = NA)
    } else if (input$PROM_type == "categorical" & input$Anchor_type == "change") {
        data.frame(PGI_C = as.factor(1:4), SSID = NA)
    } else if (input$PROM_type == "continuous" & input$Anchor_type == "static" &
input$Comparison == "last") {
        data.frame(PGI_S_chg = 1:4, SSID = NA)
    } else if (input$PROM_type == "continuous" & input$Anchor_type == "static" &
input$Comparison == "baseline") {
        data.frame(PGI_S_chg_from_bl = 1:4, SSID = NA)
    } else if (input$PROM_type == "categorical" & input$Anchor_type == "static" &
input$Comparison == "last") {
        data.frame(PGI_S_chg = as.factor(1:4), SSID = NA)
    } else if (input$PROM_type == "categorical" & input$Anchor_type == "static" &
input$Comparison == "baseline") {
        data.frame(PGI_S_chg_from_bl = as.factor(1:4), SSID = NA)
    }

    boot_fun <- function(fit) predict(fit, newdata = new_data, re.form = NA)
    set.seed(123)
    boot_res <- bootMer(model, boot_fun, nsim = 1000)
    cis <- apply(boot_res$t, 2, quantile, probs = c(0.025, 0.975))

    new_data$lower_ci <- cis[1, ]
    new_data$upper_ci <- cis[2, ]
    new_data$predicted <- predict(model, newdata = new_data, re.form = NA)

    ref_idx <- which(if (input$Anchor_type == "change") new_data$PGI_C == input$reference
else {
        if (input$Comparison == "baseline") new_data$PGI_S_chg_from_bl == input$reference
else new_data$PGI_S_chg == input$reference
    })
    ref_val <- new_data$predicted[ref_idx][1]

    plot_data <- if (input$Anchor_type == "change") {
        new_data %>% mutate(PGI = PGI_C)
    } else if (input$Anchor_type == "static" & input$Comparison == "baseline") {
        new_data %>% mutate(PGI = PGI_S_chg_from_bl)
    } else {
        new_data %>% mutate(PGI = PGI_S_chg)
```

```
        }

        plot_data %>%
          mutate(diff_from_ref = predicted - ref_val,
                 lower_diff = lower_ci - ref_val,
                 upper_diff = upper_ci - ref_val) %>%
          filter(as.numeric(PGI) != input$reference) %>%
          select(PGI, diff_from_ref, lower_diff, upper_diff)
    }, striped = TRUE)
}

shinyApp(ui, server)
```

%ESTICID SAS code

```
/*******************************************************************
  %ESTICID
  Purpose            : To ESTImate Clinically Important Difference using
an anchor-based approach for longitudinal patient-reported outcome
measures (PROMs) data.
  Description         : This macro (%ESTICID) fits mixed-effects models
to estimate
                       changes in PROMs according to an anchor (e.g.,
Patient Global Impression, PGI) over time.
                       Both continuous and categorical anchor types are
supported.
                       Generating correlation statistics, scatterplots,
and forest plots of adjusted PROM changes across anchor levels.
  Parameters:
    data              = Input dataset name (MUST)
    id                = Subject/participant ID variable (MUST)
    time              = Time variable for repeated measurements (MUST)
    anchor            = Anchor variable (e.g., PGI, CGI) (MUST)
    prom              = PROM variable to evaluate (MUST)
    categoric_anchor   = Y/N: Is anchor categorical? (MUST)
                       Y = Use LSMeans approach
                       N = Use continuous linear estimates
    reference          = Reference level for "no change"
    covstruct          = Covariance structure (default=UN)
                       Options: UN, CS, AR(1), VC, TOEP, FA(n)
    improve_dir        = Direction of improvement (default=1)
                       1  = Higher scores for anchor= improvement
(maximize)
                       -1 = Lower scores for anchor= improvement (minimize)
  Output Datasets     :
    cider_estimates = Model estimates
    cider_corr     = Correlation results

  Output Graphics     :
    1. Scatter plot with regression line (anchor vs PROM change)
    2. Within-group forest plot (mean changes by anchor level)
    3. Between-group forest plot (differences from reference)

  Notes:
    - Calculates delta values from baseline
    - Missing baseline anchor imputed as zero
    - Baseline observations excluded from modeling
```

```
    - Requires SAS 9.4+ with ODS Graphics enabled

  Example Usage:
    %CIDER(data=mydata, id=patid, time=visit, anchor=pgic,
prom=pain_score,
           categoric_anchor=Y, reference=4, improve_dir=-1);

*********************************************************************/

%macro ESTICID(
  data=,
  id=,
  time=,
  anchor=,
  prom=,
  categoric_anchor=,
  reference=,
  covstruct=UN,
  improve_dir=1
) / minoperator mindelimiter=' ';


/*=================================================================
     SECTION 1: PARAMETER VALIDATION AND INITIALIZATION

=================================================================*/

    %local _error_flag prom_var anchor_var anchor_label anchor_class;
    %local c0 c1 c2 c3;
    %let _error_flag=0;

    /* Validate required parameters */
    %if %sysevalf(%superq(data)=,boolean) %then %do;
       %put ERROR: [CIDER] Parameter DATA is required.;
       %let _error_flag=1;
    %end;
    %if %sysevalf(%superq(id)=,boolean) %then %do;
       %put ERROR: [CIDER] Parameter ID is required.;
       %let _error_flag=1;
    %end;
    %if %sysevalf(%superq(time)=,boolean) %then %do;
       %put ERROR: [CIDER] Parameter TIME is required.;
       %let _error_flag=1;
    %end;
```

```
    %if %sysevalf(%superq(anchor)=,boolean) %then %do;
        %put ERROR: [CIDER] Parameter ANCHOR is required.;
        %let _error_flag=1;
    %end;
    %if %sysevalf(%superq(prom)=,boolean) %then %do;
        %put ERROR: [CIDER] Parameter PROM is required.;
        %let _error_flag=1;
    %end;
    %if %sysevalf(%superq(categoric_anchor)=,boolean) %then %do;
        %put ERROR: [CIDER] Parameter CATEGORIC_ANCHOR is required (Y or
N).;
        %let _error_flag=1;
    %end;

    /* Check if dataset exists */
    %if not %sysfunc(exist(&data)) %then %do;
        %put ERROR: [CIDER] Dataset &data does not exist.;
        %let _error_flag=1;
    %end;

    /* Validate categoric_anchor value */
    %if %upcase(&categoric_anchor) ne Y and %upcase(&categoric_anchor)
ne N %then %do;
        %put ERROR: [CIDER] CATEGORIC_ANCHOR must be Y or N.;
        %let _error_flag=1;
    %end;

    /* Exit if errors found */
    %if &_error_flag=1 %then %do;
        %put ERROR: [CIDER] Macro terminated due to parameter errors.;
        %return;
    %end;

    /* Initialize internal variable names */
    %let prom_var=Chg_PROM;
    %let anchor_var=CID_level;
    %let anchor_label=Anchor;
    %let anchor_class=;

    /* Set intelligent defaults for reference based on anchor type */
    %if %sysevalf(%superq(reference)=,boolean) %then %do;
        %if %upcase(&categoric_anchor)=Y %then %let reference=4;
        %else %let reference=0;
    %end;
```

```
    /* Validate improve_dir */
    %if &improve_dir ne 1 and &improve_dir ne -1 %then %do;
        %put WARNING: [CIDER] IMPROVE_DIR should be 1 or -1. Setting to
1.;
        %let improve_dir=1;
    %end;

    /* Color scheme for visualizations */
    %let c0=cxBDBDBD;  /* No change / gray */
    %let c1=cxF2C94C;  /* Minimal / yellow */
    %let c2=cxF2994A;  /* Moderate / orange */
    %let c3=cxEB5757;  /* Marked / red */

    %put NOTE: ========================================;
    %put NOTE: [CIDER] Macro Execution Started;
    %put NOTE: ========================================;
    %put NOTE: Data            = &data;
    %put NOTE: ID              = &id;
    %put NOTE: Time            = &time;
    %put NOTE: Anchor          = &anchor;
    %put NOTE: PROM            = &prom;
    %put NOTE: Categoric       = &categoric_anchor;
    %put NOTE: Reference       = &reference;
    %put NOTE: Covariance      = &covstruct;
    %put NOTE: Improve Dir     = &improve_dir;
    %put NOTE: ========================================;


/*=================================================================
     SECTION 2: DATA PREPARATION

=================================================================*/

    ods graphics / attrpriority=none;

    /* Sort data by ID and time */
    proc sort data=&data out=_cider_sorted;
        by &id &time;
    run;

    /* Calculate changes from baseline */
    data _cider_changes;
        set _cider_sorted;
```

```
      by &id;

      retain base_prom base_anchor;

      /* Store baseline values */
      if first.&id then do;
         base_prom   = coalesce(&prom., 0);
         base_anchor = coalesce(&anchor., 0);
      end;
      /* Calculate changes for follow-up visits */
      else do;
         Chg_PROM   = &prom. - base_prom;
         Chg_anchor = &anchor. - base_anchor;
         CID_level  = (Chg_anchor - &reference.) * &improve_dir.;
         output;
      end;

      label
         Chg_PROM = "Change in PROM from Baseline"
         Chg_anchor = "Change in Anchor from Baseline"
         CID_level = "Standardized CID Level";
   run;

   /* Check if we have data after baseline exclusion */
   %let dsid=%sysfunc(open(_cider_changes));
   %let nobs=%sysfunc(attrn(&dsid,nobs));
   %let rc=%sysfunc(close(&dsid));

   %if &nobs=0 %then %do;
      %put ERROR: [CIDER] No follow-up observations found after baseline
exclusion.;
      %put ERROR: [CIDER] Check that your TIME variable has multiple
values per ID.;
      %return;
   %end;

   %put NOTE: [CIDER] Processing &nobs follow-up observations.;


/*=====================================================================
     SECTION 3: CORRELATION ANALYSIS

=====================================================================*/
```

```
    %put NOTE: [CIDER] Running correlation analysis...;

    ods output PearsonCorr=_cider_corr;
    proc corr data=_cider_changes nosimple;
        var CID_level &prom_var.;
    run;
    ods output close;

    /* Save correlation results */
    data cider_corr;
        set _cider_corr;
        label
            Variable = "Variable Name"
            CID_level = "Correlation with CID Level"
            Chg_PROM = "Correlation with PROM Change";
    run;


/*====================================================================
     SECTION 4: VISUALIZATION - SCATTER PLOT

====================================================================*/

    %put NOTE: [CIDER] Creating scatter plot...;

    title "Anchor vs PROM Change - Regression Analysis";
    proc sgplot data=_cider_changes;
        reg x=CID_level y=&prom_var. /
            cli
            lineattrs=(color=&c3 thickness=2)
            clm;
        scatter x=CID_level y=&prom_var. /
            markerattrs=(symbol=circlefilled color=&c0 size=8)
            transparency=0.3;
        xaxis label="Anchor (Standardized CID Level)"
              labelattrs=(weight=bold size=11)
              valueattrs=(weight=bold);
        yaxis label="Change in PROM from Baseline"
              labelattrs=(weight=bold size=11)
              valueattrs=(weight=bold);
    run;
    title;
```

```
/*=====================================================================
     SECTION 5: MIXED MODEL - CONTINUOUS ANCHOR

=====================================================================*/

   %if %upcase(&categoric_anchor) ne Y %then %do;
      %put NOTE: [CIDER] Fitting mixed model for continuous anchor...;

      ods output estimates=_cider_est_pgi;
      proc mixed data=_cider_changes;
         class &id. &time.;
         model &prom_var. = CID_level / solution ddfm=kr;
         repeated &time. / subject=&id. type=&covstruct. r rcorr;

         /* Within-group estimates at each CID level */
         estimate "Within: CID=0" intercept 1 / cl;
         estimate "Within: CID=1" intercept 1 CID_level 1 / cl;
         estimate "Within: CID=2" intercept 1 CID_level 2 / cl;
         estimate "Within: CID=3" intercept 1 CID_level 3 / cl;

         /* Between-group differences from reference */
         estimate 'Between: CID=1' intercept 0 CID_level 1 / cl;
         estimate 'Between: CID=2' intercept 0 CID_level 2 / cl;
         estimate 'Between: CID=3' intercept 0 CID_level 3 / cl;
      run;
      ods output close;

      /* Prepare within-group data for plotting */
      data _cider_graph_within;
         set _cider_est_pgi(where=(index(label,"Within:") > 0));

         /* Extract CID level from label */
         flag = input(scan(label,-1,"="), 8.);
         flagc = strip(put(flag, 1.));

         label
            flag = "CID Level (Numeric)"
            flagc = "CID Level (Character)"
            estimate = "Estimated Mean Change"
            lower = "Lower 95% CI"
            upper = "Upper 95% CI";
      run;

      /* Prepare between-group data for plotting */
```

```
    data _cider_graph_between;
        set _cider_est_pgi(where=(index(label,"Between:") > 0));

        /* Extract CID level from label */
        flag = input(scan(label,-1,"="), 8.);
        flagc = strip(put(flag, 1.));

        label
            flagc = "CID Level"
            estimate = "Mean Difference"
            lower = "Lower 95% CI"
            upper = "Upper 95% CI";
    run;

%end;


/*==================================================================
     SECTION 6: MIXED MODEL - CATEGORICAL ANCHOR

==================================================================*/

%if %upcase(&categoric_anchor) = Y %then %do;
    %put NOTE: [CIDER] Fitting mixed model for categorical anchor...;

    ods output diffs=_cider_diff_pgi lsmeans=_cider_lsmeans_pgi;
    proc mixed data=_cider_changes;
        class &id. &time. CID_level(ref="0");
        model &prom_var. = CID_level / solution ddfm=kr;
        repeated &time. / subject=&id. type=&covstruct. r rcorr;
        lsmeans CID_level / cl diff;
    run;
    ods output close;

    /* Prepare within-group data */
    data _cider_graph_within;
        set _cider_lsmeans_pgi(where=(CID_level in (0,1,2,3)));

        flag = CID_level;
        flagc = strip(put(flag, 1.));

        label
            flag = "CID Level (Numeric)"
            flagc = "CID Level (Character)"
```

```
            estimate = "LS Mean"
            lower = "Lower 95% CI"
            upper = "Upper 95% CI";
    run;

    /* Prepare between-group data */
    data _cider_graph_between;
        set _cider_diff_pgi(where=(_CID_level=0 and CID_level in
(1,2,3)));

        flag = CID_level;
        flagc = strip(put(flag, 1.));

        label
            flagc = "CID Level"
            estimate = "Mean Difference from Reference"
            lower = "Lower 95% CI"
            upper = "Upper 95% CI";
    run;

%end;

/* Save estimates to output dataset */
%if %upcase(&categoric_anchor) = Y %then %do;
    data cider_estimates;
        set _cider_lsmeans_pgi _cider_diff_pgi;
    run;
%end;
%else %do;
    data cider_estimates;
        set _cider_est_pgi;
    run;
%end;


/*==================================================================
     SECTION 7: FOREST PLOT TEMPLATES AND RENDERING

==================================================================*/

%put NOTE: [CIDER] Creating forest plots...;

/* Within-group forest plot template */
proc template;
```

```
      define statgraph cider_within_forest;
         begingraph / border=false;

            discreteattrmap name="cid_colors";
               value "0" / markerattrs=(color=cxBDBDBD)
lineattrs=(color=cxBDBDBD thickness=2);
               value "1" / markerattrs=(color=cxF2C94C)
lineattrs=(color=cxF2C94C thickness=2);
               value "2" / markerattrs=(color=cxF2994A)
lineattrs=(color=cxF2994A thickness=2);
               value "3" / markerattrs=(color=cxEB5757)
lineattrs=(color=cxEB5757 thickness=2);
            enddiscreteattrmap;

            discreteattrvar attrvar=grp_color var=flagc
attrmap="cid_colors";

            layout overlay /
               yaxisopts=(type=discrete
                     label="Anchor"
                     labelattrs=(weight=bold size=11)
                     tickvalueattrs=(weight=bold)
                     offsetmin=0.15
                     offsetmax=0.15
                     discreteopts=(tickvaluelist=("0" "1" "2" "3")
                           tickdisplaylist=("No Change"
                                 "Minimal Improvement"
                                 "Moderate
Improvement"
                                 "Marked
Improvement")))
               xaxisopts=(label="Mean Change in PROM"
                     labelattrs=(weight=bold size=11)
                     tickvalueattrs=(weight=bold)
                     offsetmin=0.05
                     offsetmax=0.05)
               border=false;

               referenceline x=0 / lineattrs=(pattern=dash
color=gray);

               scatterplot y=flagc x=estimate / group=grp_color
                  markerattrs=(symbol=circlefilled size=10)
                  xerrorlower=lower
```

```
                    xerrorupper=upper
                    errorbarcapscale=0.75
                    name='scatter';

                endlayout;
            endgraph;
        end;
    run;

    title "CID Within-Group";
    proc sgrender data=_cider_graph_within
template=cider_within_forest;
    run;
    title;

    /* Between-group forest plot template */
    proc template;
        define statgraph cider_between_forest;
            begingraph / border=false;

                discreteattrmap name="cid_colors_b";
                    value "1" / markerattrs=(color=cxF2C94C)
lineattrs=(color=cxF2C94C thickness=2);
                    value "2" / markerattrs=(color=cxF2994A)
lineattrs=(color=cxF2994A thickness=2);
                    value "3" / markerattrs=(color=cxEB5757)
lineattrs=(color=cxEB5757 thickness=2);
                enddiscreteattrmap;

                discreteattrvar attrvar=grp_color_b var=flagc
attrmap="cid_colors_b";

                layout overlay /
                    yaxisopts=(type=discrete
                            label="Anchor"
                            labelattrs=(weight=bold size=11)
                            tickvalueattrs=(weight=bold)
                            offsetmin=0.15
                            offsetmax=0.15
                            discreteopts=(tickvaluelist=("1" "2" "3")
                                       tickdisplaylist=("Minimal
Improvement"
                                                   "Moderate
Improvement"
```

```
                                                         "Marked
Improvement")))
                    xaxisopts=(label="Mean Difference from Reference"
                              labelattrs=(weight=bold size=11)
                              tickvalueattrs=(weight=bold)
                              offsetmin=0.05
                              offsetmax=0.05)
                    border=false;

                    referenceline x=0 / lineattrs=(pattern=dash
color=gray);

                    scatterplot y=flagc x=estimate / group=grp_color_b
                        markerattrs=(symbol=circlefilled size=10)
                        xerrorlower=lower
                        xerrorupper=upper
                        errorbarcapscale=0.75
                        name='scatter';

                endlayout;
            endgraph;
        end;
    run;

    title "CID Between-Group";
    proc sgrender data=_cider_graph_between
template=cider_between_forest;
    run;
    title;


/*===================================================================
      SECTION 8: CLEANUP

===================================================================*/

    %put NOTE: [CIDER] Cleaning up temporary datasets...;

    proc datasets library=work nolist nowarn;
        delete _cider_: / memtype=data;
    quit;

    %put NOTE: ========================================;
    %put NOTE: [CIDER] Macro Execution Completed;
```

```sas
    %put NOTE: Output datasets: cider_estimates, cider_corr;
    %put NOTE: ==========================================;

%mend ESTICID;


/*=============================================================
  USAGE EXAMPLES
=============================================================*/

/*
Example 1: Continuous anchor (PGI-C scale 1-7, where 4=no change)
Lower scores of anchor = improvement, so improve_dir = -1
*/

%ESTICID(
    data=supp1,
    id=ssid,
    time=time,
    anchor=pgi_c,
    prom=prom,
    categoric_anchor=N,
    reference=4,
    covstruct=CS,
    improve_dir=-1
);

/*
Example 2: Categorical anchor (change score where 4=no change)
Lower scores of anchor = improvement
*/

%ESTICID(
    data=supp1,
    id=ssid,
    time=time,
    anchor=pgi_c,
    prom=prom,
    categoric_anchor=Y,
    reference=4,
    covstruct=UN,
    improve_dir=1
);
```

```
PROC IMPORT
    DATAFILE="D:\supp1.xls"
    OUT=supp1
    DBMS=EXCEL
    REPLACE;
    GETNAMES=YES;
RUN;
```
